# An Unusual Case of Chronic Kidney Disease with Mediastinal Lymphadenopathy

**DOI:** 10.7759/cureus.2646

**Published:** 2018-05-18

**Authors:** Abhishek Thandra, Daniel Munley, Jonathan Gapp, Sunil Jagadesh

**Affiliations:** 1 Internal Medicine, Creighton University Medical Center, Omaha, USA; 2 Medical Student, Creighton University School of Medicine, Omaha, USA; 3 Nephrology, Creighton University Medical Center, Omaha, USA

**Keywords:** chronic kidney disease, gomerular interstitial nephritis, lymphadenopathy

## Abstract

Granulomatous interstitial nephritis (GIN) is a rare histological diagnosis seen in less than 1% of native renal biopsies. This case report describes a 37-year-old male with chronic kidney disease (CKD) and mediastinal lymphadenopathy. The kidney biopsy showed granulomatous interstitial nephritis with mild interstitial fibrosis and variable tubular atrophy, as well as a lymph node biopsy with non-caseating granulomas. All the etiologies for non-caseating granulomas, such as infections (mycobacterial, fungal, bacterial, viral infections), hypersensitivity reactions, autoimmune disorders, and granulomatosis with polyangiitis), were initially considered and later excluded as the workup was negative. The patient was unresponsive to a trial of steroids and continued to be on dialysis. This case highlights the importance of obtaining a kidney biopsy in patients with progressive renal dysfunction without traditional risk factors such as hypertension and diabetes.

## Introduction

Chronic kidney disease (CKD) is a growing public health issue worldwide with the number of patients increasing each year. Over 10% of the population worldwide is affected by CKD, and about two million people currently receive treatment with dialysis or a kidney transplant [1]. According to the National Institute of Health, the adjusted incidence of CKD specifically in the United States has been declining since its peak in 2006; however, the prevalence of CKD in the general population of the United States remains at approximately 14% [2]. Around 661,000 Americans qualify as having kidney failure, and of those patients, 468,000 receive dialysis. While hypertension and diabetes are the common causes of CKD, there are many other underlying conditions which can lead to both primary and secondary kidney disease. It is important that these less common etiologies be considered in the differential for atypical patients, such as the one discussed in this report. Granulomatous interstitial nephritis (GIN) is a rare histological diagnosis that accounts for only 0.5% to 0.9% of the native renal biopsies and 0.6% of renal transplant biopsies [3].

## Case presentation

A 37-year-old male with a past medical history of hypothyroidism and recent contact with a meningitis patient was transferred to our tertiary care center after presenting to a previous hospital for a one-day history of altered mental status, headache, and presumed lymphocytic predominant meningitis. At the previous institution, the patient was given 2 gm of ceftriaxone, as well as an opioid medication for his headache. A lumbar puncture performed there showed a negative gram stain, an elevated white blood cell count of 19 x 10/µL with 89% lymphocytes, an elevated protein of 54 mg/dl, and an equivocal glucose of 65 mg/dl. Cultures from this initial spinal fluid sample were pending at the time of presentation with ensuing results showing no growth at three and four days. On general physical examination, he had generalized weakness, the vitals were stable, and the systemic examination was completely normal without any neck rigidity or pain, skin rash, palpable lymph nodes, or hepatosplenomegaly.

Serum laboratory studies showed a normocytic anemia with hemoglobin of 8.4 g/dL (normal range: 13.5 - 17 g/dL) and a normal white blood cell count with differential. His initial serum basic metabolic panel (BMP), however, was concerning for azotemia with a blood urea nitrogen (BUN) of 98 mg/dl (normal range: 8 - 24 mg/dL) and a creatinine of 11.90 mg/dl (normal range: 0.6 - 1.3 mg/dL) without a known baseline creatinine. The patient’s estimated glomerular filtration rate (GFR) at that time was decreased to 6 mL/min/1.73m^2^. The initial serum metabolic panel additionally showed a metabolic acidosis with an elevated anion gap of 19 mEq/L and a bicarbonate of 18 mmol/L with a repeat BMP later that day showing similar values. Urinalysis on the day of admission showed an elevated protein of 30 mg/dl with a small amount of blood, and random urine protein was elevated at 107 mg/dl. Random urine creatinine was 42 mg/dl, with a urine protein to creatinine ratio of approximately 2.5 gm/gm of creatinine. The patient’s remaining labs, including serum lactic acid, serum uric acid, phosphorus, and magnesium, were unremarkable. Human immunodeficiency virus (HIV) testing was negative.

Considering his progressively worsening renal function, a renal ultrasound and nephrology consult were obtained. The ultrasound showed a 1 cm non-obstructive renal calculus in the inferior pole of the left kidney with no evidence of hydronephrosis and a normal-appearing right kidney.

**Figure 1:**
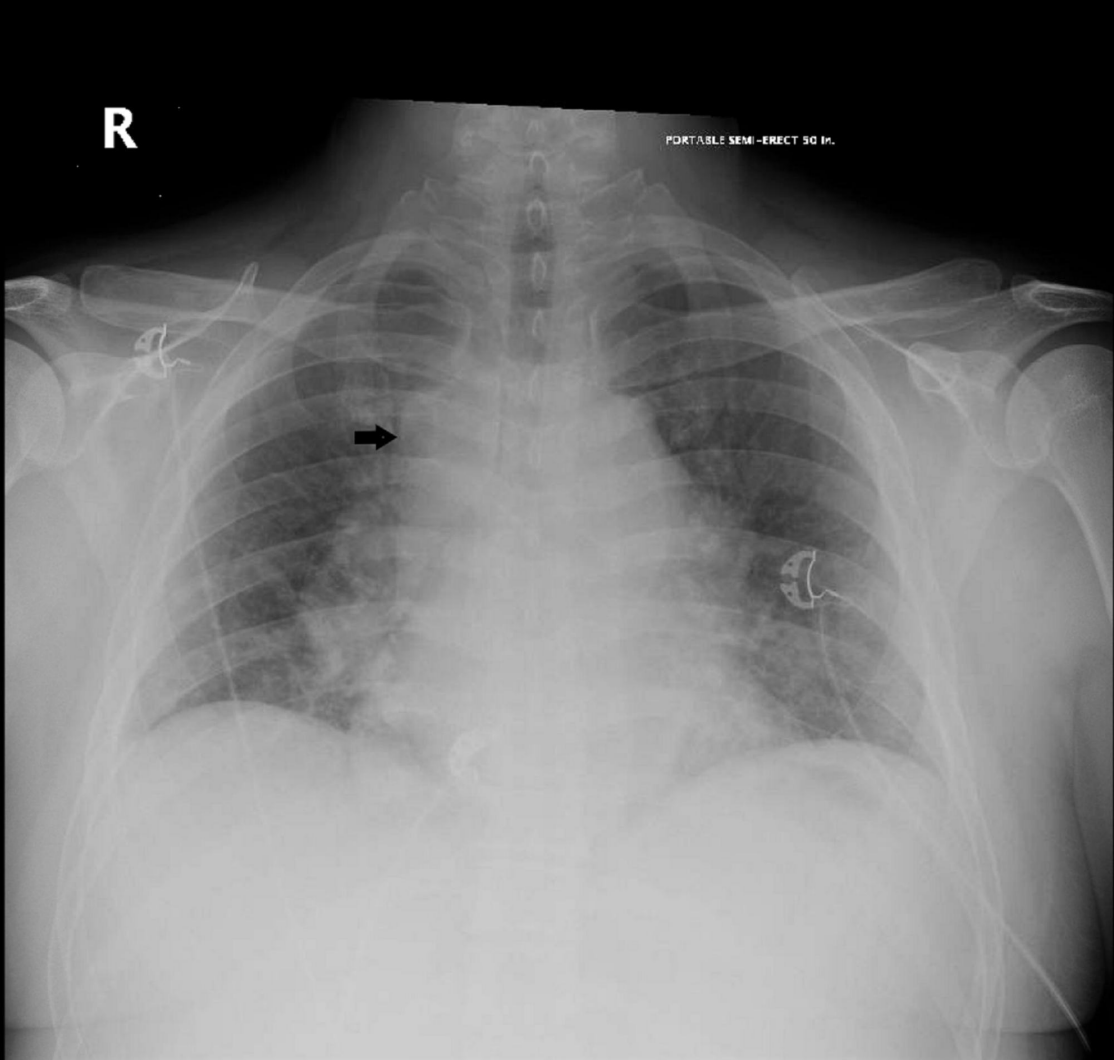
Chest x-ray with ill-defined opacity overlying the mediastinum (black arrow)

**Figure 2:**
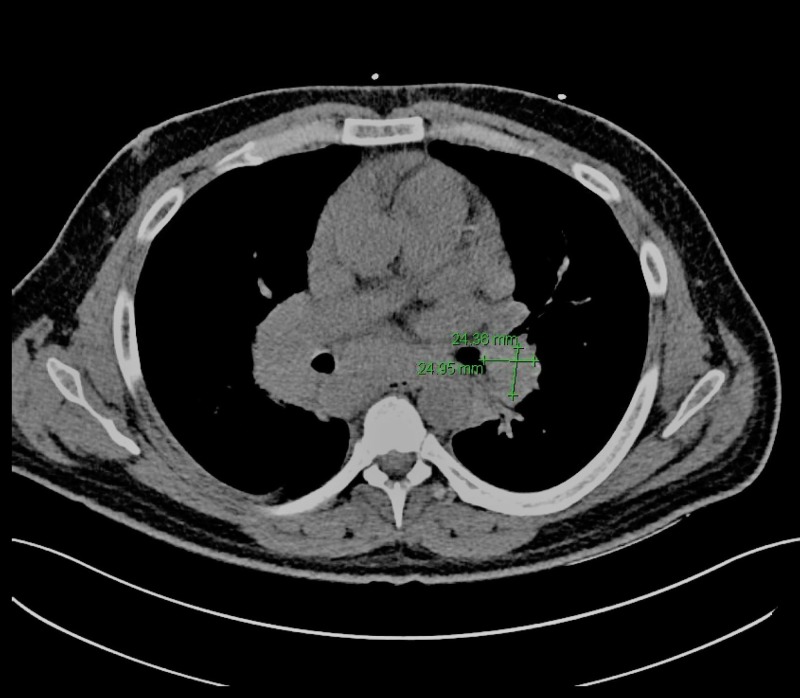
Computed tomography imaging of the chest shows enlarged left hilar lymph node measuring about 2.4 x 2.4 cm

Investigation of the patient’s altered mental status and suspected lymphocytic predominant meningitis consisted of immediate blood cultures, as well as a computed tomography (CT) of the head and chest x-ray completed on the day of his admission. The head CT was unremarkable, while the chest x-ray showed an ill-defined opacity overlying the mediastinum with a differential including tumor, vascular aneurysm, foreign body, or lymphadenopathy (Figure 1). Further investigation with a chest CT showed diffuse mediastinal, bilateral hilar, and right supraclavicular lymphadenopathy concerning for lymphoma with partial compression of the hilar bronchi secondary to hilar lymphadenopathy (Figure 2). Although central nervous system (CNS) lymphoma was considered higher on the differential than meningitis, the patient was empirically started on ceftriaxone, vancomycin, and acyclovir until culture results were available. Cerebrospinal fluid obtained by a lumbar puncture at the previous hospital was sent to our institution and tested through BioFire PCR (BioFire Diagnostics, Salt Lake City, UT) against a meningitis/encephalitis panel of 14 pathogens, all of which returned as negative. The patient’s blood cultures remained negative for growth after five days. Over the next several days, the patient’s cerebrospinal fluid analysis, flow cytometry, and viral panel were negative. However, his kidney function and overall status continued to deteriorate.

Two days after admission, the patient had a right supraclavicular lymph node core biopsy and had a repeat lumbar puncture with spinal fluid analysis on the third day, both at the recommendation of the hematology-oncology consultant, to assess for possible CNS lymphoma. The repeat spinal fluid analysis was unremarkable but the lymph node biopsy showed scattered loose aggregates of histiocytes (loose granulomas) without necrosis. Per the pathology report, the loose aggregates of histiocytes did not demonstrate the tight, organized granuloma formations most commonly found in sarcoidosis; however, it was noted that the granuloma architecture could not reliably distinguish among the causes of non-caseating granulomas. Special stains did not reveal acid-fast or fungal organisms. There was no evidence of lymphoproliferative disease, and this pathology report was later found to be consistent with a previous lymph node biopsy in 2011 which was done for asymptomatic lymphadenopathy and had shown non-necrotic granulomatous tissue. The diagnosis of CNS lymphoma was effectively excluded with these findings; however, no reliable diagnosis for this granulomatous change could be made.

**Figure 3:**
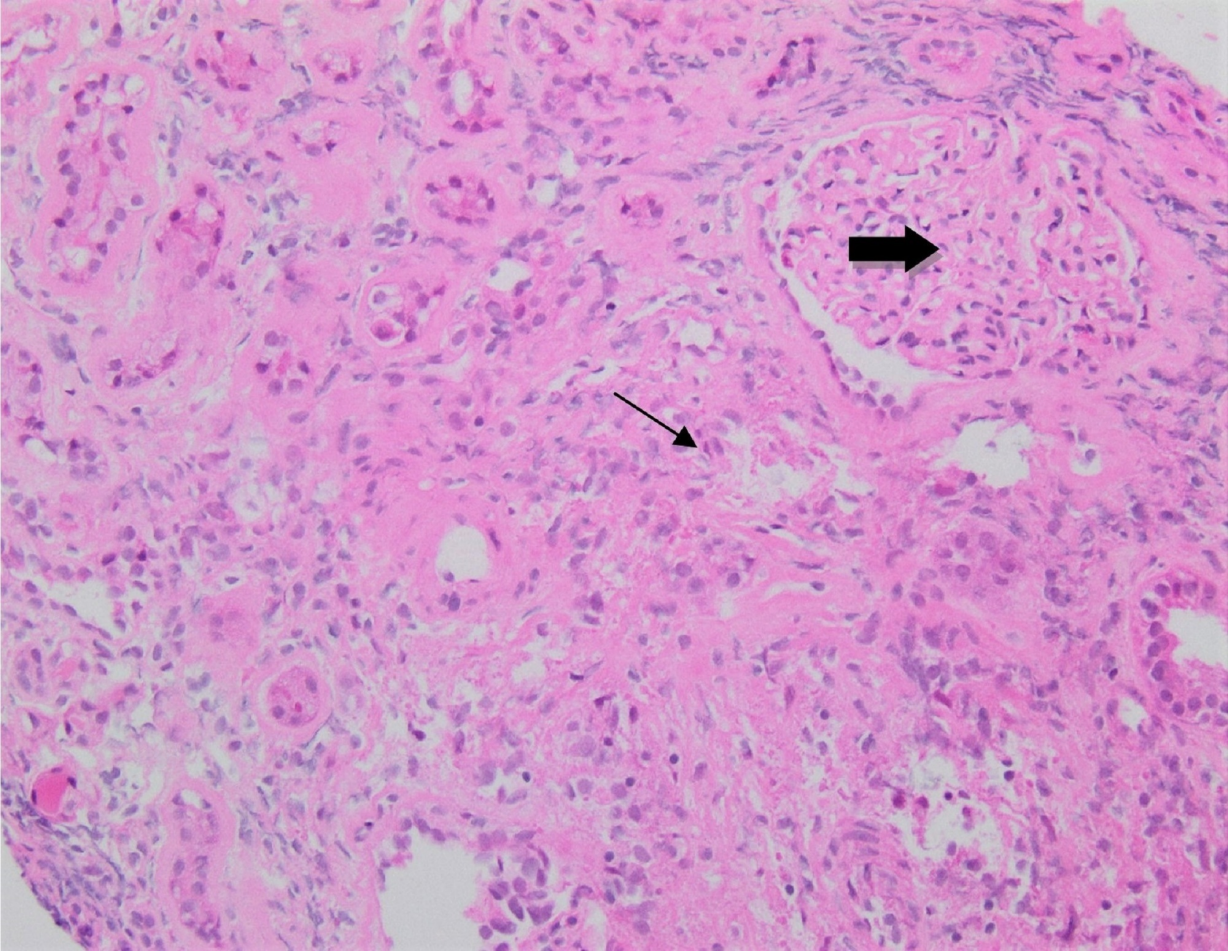
Kidney biopsy Histology shows granulomatous interstitial nephritis with mild interstitial fibrosis (thin arrow) and interstitial inflammation and fibrosis within the glomeruli (thick arrow)

During this period, the patient’s kidney function and azotemia continued to worsen with the BUN reaching 133 mg/dl and creatinine reaching 10.40 mg/dl three days after admission. His metabolic acidosis with an elevated anion gap continued to rise as well, and three days after admission, the patient was started on hemodialysis for symptoms suggestive of uremia. A kidney biopsy was also obtained, considering the worsening renal function, which displayed granulomatous, interstitial nephritis with mild, interstitial fibrosis and variable tubular atrophy (Figure 3). The absence of crescents on the renal biopsy placed glomerulonephritis low on the differential. There was no obvious thickening of the glomerular basement membrane and the periodic acid-Schiff (PAS) stain showed only mild atrophic change. As in the patient’s lymph node biopsy, special stains for acid-fast bacilli and Grocott's methenamine silver (GMS) stain for fungus were negative for organisms within the poorly-formed granulomas. The pathologist determined that these ill-defined granulomas were not typical of sarcoidosis. The differential diagnosis for granulomatous change was expanded to include the possibility of drug-related granulomatous interstitial nephritis (GIN) or granulomatosis with polyangiitis (GPA). A diagnosis of GPA, although entertained, was unlikely in the absence of crescents or vasculitic changes. An antineutrophil cytoplasmic autoantibody (ANCA) panel was negative and the serum angiotensin-converting enzyme level was normal. The patient was tested for levels of anti-double-stranded deoxyribonucleic acid (DNA) antibody, anti-scleroderma antibody, cyclic citrullinated peptide (CCP) antibody, rheumatoid factor, and Sjogren’s syndrome-A extractable nuclear antibody, all of which were negative.

Over the course of his stay, the patient received five sessions of hemodialysis and his labs, including his azotemia and anion gap, improved immensely on this therapy. Upon discharge, 10 days after admission, the patient’s azotemia and metabolic acidosis had resolved completely and he had returned to baseline mental status. The anemia he had on admission, presumably due to chronic disease, had also resolved. The renal consultants recommended that he continue hemodialysis three days per week and be discharged on prednisone, 40 mg daily, to be tapered 10 mg every two weeks.

After the one-month trial of prednisone, he was seen in the clinic and continued to require dialysis as kidney function did not improve.

## Discussion

The annual data report on CKD published by the National Institute of Health identifies age as the greatest predisposing risk factor for chronic kidney disease. The age group of 20-39 years has been shown to have the lowest prevalence of this condition in adult men, further placing this patient as an outlier [4]. The combined pathology reports from the patient’s left kidney biopsy and right supraclavicular lymph node core biopsy both show granulomatous changes, making it likely that the patient has a systemic granulomatous disease underlying his kidney failure.

The differential for non-caseating granulomas is wide and includes mycobacteria, fungi, bacteria, sarcoidosis, hypersensitivity reactions, parasites, rickettsia, viruses, chlamydia, foreign body reactions, and GPA. Because stains for acid-fast bacilli and GMS stain for fungus were negative, it is unlikely that either mycobacteria or fungi were the cause. The patient’s negative blood cultures and lack of clinical signs of infection make it doubtful that his condition had an infectious etiology that might have spread either hematogenously or lymphatically. GPA was a possible diagnosis because it may manifest as glomerulonephritis with mediastinal lymphadenopathy as a nonspecific finding [5]. However, while non-caseating granulomas were present in this patient, there was no evidence of vasculitis or of airway involvement at the time of presentation, a common hallmark of the disease. Moreover, the ANCA panel was negative. Another possible underlying condition is sarcoidosis, which can present as GIN [6]. Previously, a case series of seven patients with GIN without extrarenal sarcoidosis was conducted in which five patients responded to steroids and two required dialysis [7]. The official pathology report in our case stated that the non-caseating granulomas seen in the patient’s kidney biopsy were not typical of sarcoidosis. These findings, along with non-response to steroids, made sarcoidosis unlikely [3]. However, the report was not able to make a definitive alternative diagnosis. Ultimately, a diagnosis of idiopathic granulomatous interstitial nephritis was made.

## Conclusions

Chronic kidney disease in a young patient, as seen in this case, is uncommon, especially in the absence of traditional risk factors, such as hypertension or diabetes mellitus. The non-caseating granulomas seen on renal biopsy and enlarged right supraclavicular lymph node made for an even more uncommon presentation. In the literature, most patients with granulomatous interstitial nephritis are responsive to steroids; this was not true in our case. This case highlights the importance of obtaining a kidney biopsy when pursuing an etiology of progressive renal dysfunction in the absence of traditional risk factors. 
